# Mr. Quain's Lectures on Anatomy and Physiology

**Published:** 1831-01-01

**Authors:** 


LXII.

TWO LECTURES ON THE STUDY OF ANATOMY AND PHYSIOLOGY. Delivered at the Opening of the Session 1830, in the Medical School, Aldersgate Street. By JONES QUAIN, M.B.

WE do not intend to review nor to criticise these Lectures. Mr. Quain is very favourably known to the profession as the author of a valuable work upon anatomy, and to young men as an anatomical teacher. The present lectures do no discredit to his talents or his reputation. The moral tone which animates them is highly commendable in a teacher, and the interesting manner in which the subjects are handled, is calculated to lead his auditors to embark with zeal in studies which can wear so pleasing a garb. The following passage will exhibit a specimen of the whole.

"The personal I is confessed a permanent being; every individual acts as if he were one and identical; and such he is invariably considered by others, notwithstanding the admitted fact, that the material components of his body are subject to a perpetual mutation; for, over this ceaseless cycle of change presides that power, which altogether suspends the ordinary play of affinities in the first moments of fœtal existence, modifies and controls them during the succeeding stages of life, and allows them to come into action, only when it is withdrawn at death. 'I had rather,' says Bacon, 'believe all the fables of the Legend, the Talmud, and the Koran, than that this universal frame is without a mind. When the mind of man looketh to second causes scattered, it may sometimes rest on them, and go no farther; but when it beholdeth the chain of them confederate and linked together, it must needs flee to Providence and to Deity.' How strangely then do those men argue, who contend that all the phenomena of living beings, and all the functions which they perform, are results—the necessary results of their organization; and that their structure is produced by an aggregation of particles, according to the laws of chemical attraction. We have seen, however, that such is not the rule of their formation; so far from it, they are formed by a process the very reverse of this; which is a conclusive evidence that there is some other power at work, besides that of attraction. But, were we, for a moment, to admit that the form and structure of organized bodies are determined by attraction, then we could have no grounds for expecting to find evidence of design or forethought in their conformation. This at once prompts us to enquire, (and surely it is an interesting subject of enquiry) whether they do not exhibit incontestible evidences of both, in whatever point of view we examine their habits and capabilities, or investigate their structure.

It is a favorite opinion with many that all our knowledge is derived from the senses; as well might it be said that all arts and manufactures are derived from the doors and windows of the houses, into which the raw materials are brought to be subjected to the skill and dexterity of the workmen. Again, as our senses exist before we have acquired any experience, we have sufficient grounds for questioning another assertion, which is frequently put forth, namely, that all knowledge comes from experience. There is a sort of knowledge which is prior to experience, and acts quicker than reason, and which exhibits itself for the most part in prompting measures for self-preservation. Thus young animals seek the breast from which their nutriment is derived; and, in after-life, the different tribes of living beings select different sorts of substances for their food: some feed on herbs, and every part of their conformation marks them to be fitted and intended for digesting that kind of food. Others live on animal substances, and as we saw yesterday, when examining the structure of carnivorous animals, the conformation of their teeth, jaws, stomach, limbs, adapt them for the habits that have been impressed on them. Some become torpid during winter, and choose places of security whilst in that state; others, as the swallow, enjoy a perpetual summer, by migrating from one country to another, and their conformation enables them to fulfil their destination. The bee and the wasp lay up stores for winter, and, strange to say, the comb which the bee builds is always placed vertically, that of the wasp, horizontally. Moreover, the cells are all constructed on strictly geometrical principles; for each of them is a hexagon, terminated by a pyramidal base. In the execution of their work they give a practical solution of a very difficult problem: 'A quantity of wax being given to form out of it, similar and equal cells of a determinate capacity, but at the same time so arranged, collectively, as to occupy the smallest possible space, whilst each individual cell possesses the largest possible area in proportion to the quantity of matter employed.' If they were cylindrical, vacant spaces must exist between each three contiguous cells; if they were square or triangular, they would require more material and be altogether unsuited to the form of the bee's body.

Is it from instruction — is it from their senses — is it from experience, that these creatures execute their work with the precision and method of the most accomplished artists? No one, I believe, would answer in the affirmative; each group of living things has its special aptitudes, its peculiar habits.

Dente lupus, cornu taurus petit; unde nisi intus

Monstratum?"

We wish Mr. Quain every success which assiduity and honest ambition deserve.

---

